# Identification and Discrimination of Brands of Fuels by Gas Chromatography and Neural Networks Algorithm in Forensic Research

**DOI:** 10.1155/2016/6758281

**Published:** 2016-06-08

**Authors:** L. Ugena, S. Moncayo, S. Manzoor, D. Rosales, J. O. Cáceres

**Affiliations:** Department of Analytical Chemistry, Faculty of Chemical Sciences, Complutense University, 28040 Madrid, Spain

## Abstract

The detection of adulteration of fuels and its use in criminal scenes like arson has a high interest in forensic investigations. In this work, a method based on gas chromatography (GC) and neural networks (NN) has been developed and applied to the identification and discrimination of brands of fuels such as gasoline and diesel without the necessity to determine the composition of the samples. The study included five main brands of fuels from Spain, collected from fifteen different local petrol stations. The methodology allowed the identification of the gasoline and diesel brands with a high accuracy close to 100%, without any false positives or false negatives. A success rate of three blind samples was obtained as 73.3%, 80%, and 100%, respectively. The results obtained demonstrate the potential of this methodology to help in resolving criminal situations.

## 1. Introduction

Classification and identification of petroleum fuels are a crucial challenge in the scientific investigation of arson and have high importance in cases of commercial, industrial, and forensic criminal acts. The crime of arson may be defined as a willful and malicious act to setting fire to a property and is considered one of the easiest crimes to commit and yet also one of the hardest to investigate [1, 2]. The liquids commonly used as accelerants in the criminal acts are gasoline, kerosene oil, or diesel due to their easy availability and low price [2, 3]. Regulations established for the analysis of ignitable liquid residues provide a common language to the field of forensic science for describing the characteristics of the different inflammable liquids as well as to classify them [4–6].

The quality control of the fuels is guaranteed by means of establishing some technical specifications that vary in different parts of the world (i.e., EN 228 in Europe, ASTM D48 14 in the USA, JIS K2202 in Japan, and IS 2796 in India) [7]. In particular, commercial or industrial adulteration with lower-price fuels is one of the most difficult situations to detect and is a serious problem in the petrochemical industry because of the similar composition of the adulterant fuels used.

The contents of oxygen and hydrocarbons such as aromatics and olefins vary in different samples that produce various kinds of petroleum derivatives with specific characteristics belonging to a particular refinery [1, 8]. The chemical composition of the crude oil depends on various factors that must be taken into account each time when performing analysis of these samples and collecting reference materials. These factors include the origin (oilfields and country), refining procedures, and the location of the distributors. Apart from these elements, another important factor is the contamination introduced in the fuels due to the residual level of the storage tank [3].

In many situations where fuels are used in criminal acts, their composition may vary significantly subjecting to the fire. Factors like air flow and high temperature affect the composition because the more volatile compounds could evaporate. Moreover, the fuels may get contaminated by biological processes such as bacterial degradation [1]. For these reasons, the analysis of the samples could become more difficult. However, there are sites on the crime scene such as the porous materials where the accelerant liquids stay unaffected and retain their original composition that can be recovered for the analysis [1, 8, 9].

Several methods have been proposed to identify and discriminate fuel samples, based on gas chromatography-mass spectrometry (GC-MS) [1–3, 8–13], as well as the determination of additives by normal-phase high-performance liquid chromatography (NP-HPLC) combined with GC-MS [14], and various spectroscopic methods such as Nuclear Magnetic Resonance (NMR), Near Infrared (NIR) spectroscopy and Attenuated Total Reflection-Fourier Transform (ATR-FTIR) spectroscopy [7, 15–19]. Although these analytical techniques are widely used in determination of the composition and the classification of the fuels, there are some drawbacks because they are time consuming and expensive for the analysis. Raman spectroscopy has also been used for the classification of gasoline; nevertheless, there are not sufficiently large differences in the Raman spectra for unambiguous identification [20].

Detection and identification of fuels using GC analysis have been studied by several research groups [13, 21, 22]. The main reason for the application of GC methods to ignitable liquid residue analysis is the need for adequate separation of components comprising petroleum-based fuels [23]. Most of the discriminating information is contained in the fraction with a volatility ranging from approximately n-pentane to n-octane [13]. Many of these studies have focused on determining the fuels using chemometric procedures such as Principal Component Analysis (PCA), Linear Discriminant Analysis (LDA), Soft Independent Modeling of Class Analogy (SIMCA), and Quadratic Discriminant Analysis (QDA) [1, 3, 7–9, 15–21].

Although these studies provided acceptable results, in some cases, correct identification rate of fuels falls below 85%. Error rates in many of them are too high to classify a particular gasoline or if there are false negatives or false positives in the results. Moreover, studies where the neural networks (NN) chemometric method has been used, only the trainings of the models are made, not performing test-sets or blind samples to validate the accuracy and robustness of the method [9]. Furthermore, when the unknown samples are used, methods such as LDA are completely unable to make a correct classification. Unfortunately, many of these authors do not take into account or are unaware of the pitfalls in their results. Therefore, there is a need for more systematic studies that include new approaches to identifying and classifying brands of fuels accurately. Another studies have been performed combining NN with another techniques using different types of samples. The satisfactory results, obtained in this methodology using NN, gave us the approach to trying to analyze samples of fuels [24, 25].

Specifically, the aim of this work was to improve the recognition capacity by developing a method capable of identifying extremely similar samples that have few differences between them. Therefore, NN algorithms were combined with GC as they are capable of dealing with large and multivariate information increasing the classification capacity, due to their simple implementation and ability to generalize that makes them useful for qualitative analysis and to improve the accuracy and precision of the classification process. It was intended to develop a methodology based on GC and NN to determine exactly the point of purchase of the gasoline used in a criminal act.

## 2. Materials and Methods

### 2.1. Fuel Samples

Five brands of fuels (Repsol, Shell, British Petroleum (BP), Cepsa, and Galp) collected from fifteen different local petrol stations were studied. For each brand, three different service stations were chosen to collect a sample of gasoline and diesel. Fifteen samples of gasoline and diesel of each were analyzed. All samples were collected in December 2013 in Madrid, Spain. All samples were stored in a glass recipient at −20°C in a freezer to avoid the loss of volatile compounds. For each sample of diesel and gasoline, five chromatograms were acquired, respectively; therefore, a total of 150 chromatograms were recorded. Additionally, three blind samples were collected randomly from these service stations, labeled as “A,” “B,” and “C”. Experimental conditions were maintained the same as used for the references, recording three chromatograms for each blind sample.

### 2.2. Gas Chromatography Analysis

All analyses were performed using an Acme6000 gas chromatograph connected to a flame ionization detector (FID). The GC was equipped with a 100% polydimethylsiloxane capillary column ZB1 (30 m × 0.25 mm × 0.25 *μ*m). The sample injection volume was 1 *μ*L, delivered by syringe with a split ratio of 50 : 1. Samples were analyzed using the following chromatographic conditions: diesel samples were measured using split injection (2 : 1) at 300°C; the oven temperature was programmed from 70°C (1 min isothermal) to 300°C at 10°C/min (held for 3 min). Injector pressure was 30 psi and detector temperature was 350°C. Gasoline samples were performed using split injection (2 : 1) at 300°C; the oven temperature was ramped from 40°C (1 min isothermal) to 100°C at 15°C/min, followed by a ramp to 300°C at 50°C/min (hold time of 3 min), giving a total run time of 28 min of each chromatogram. Injector pressure was 30 psi and detector temperature was 350°C. In this work, as a pure sample was used, the chromatographic conditions of diesel and gasoline are different due to the different time of retention of the compounds. Diesel samples are more volatile than gasoline samples and it was necessary for obtaining optimum chromatograms to use different gradients of temperature.

### 2.3. Neural Networks Model

Home-made neural networks software specifically designed to deal with the discrimination of different brands of fuels was developed. The NN models were based on a multilayer perceptron, feedforward, supervised network that consisted of several neurons (information processing units) arranged in two or more layers receiving information from all of the neurons of the previous layer. The connections are controlled by a weight that modulates the output from the neuron before inputting its numerical content into a neuron in the next layer.

The NN topology consists of three layers (input, hidden, and output), which is widely used to model systems with a similar level of complexity [26]. In particular, the input layer consisted of 1440 nodes (intensity of chromatogram within selected range of retention time). The number of neurons in the hidden layer was set to 20. The output layer was comprised of *J* neurons (where *J* is number of reference samples used) for estimating the similarity between the reference sample (reference chromatogram) and the testing sample chromatogram, denoted hereafter as test-set.

The process that optimizes the weights, that is, the learning or training process was based on a back-propagation algorithm [27–29]. The inputs from each neuron are added by an activation function, and the result is transformed by a transfer function that limits the amplitude of the neuron output. In this work, the hyperbolic tangent sigmoid function was used as the NN transfer function. Every NN model was estimated using Matlab software (Mathworks, 2010a).

Because the NN is a supervised method, in order to optimize the weight matrix, it is necessary to use input and output data that adequately characterized the system to be modeled. The chromatographic data of the training library was randomly divided as part of the training process into two subsets: 75% for training and 25% for self-validation of the model. Two NN models (diesel and gasoline) were developed. Once the training and self-validation process was carried out, the models were validated with test-set, with chromatograms being not used in the training process. The identification process was based on the ability of the NN to detect the degree of similarity between the unknown chromatogram and each of the reference chromatograms used in the training process. During the training process, each brand of fuel was associated with an identification number in the output layer. Thus, a perfect identification is obtained if the output from the NN model for the test samples of the same brand of fuel matched the identification number assigned to the brand used to train the model. Zero identification number was always used to indicate no match at all. NN training was achieved by applying the back-propagation algorithm based on the conjugate gradient method [30], one of the general-purpose second-order techniques that helps minimize the goal functions of several variables. Second order indicates that such method uses the second derivatives of the error function, whereas a first-order technique, such as standard back propagation, uses only the first derivatives. To determine when the training should be stopped, an early stopping criteria based on performance improving (error rate) of the validation set was used [31]. The number of epochs was not relevant in this case as verification mean square error (MSE), as defined in (1), was taken into account to avoid an overfitting of the NN model. For this purpose, the learning process was repeated until MSE was decreased:

(1) $$\begin{array}{c} \text{MSE}=\frac{1}{N}\overset{N}{\underset{k}{\sum }}{\left(\;r_{k}-y_{k}\;\right)}^{2}, \end{array}$$

where *N*, *r*_*k*_, and *y*_*k*_ are the number of input data, target output, and the response from each output neuron, respectively. A detailed description of the calculation process is provided in the literature [28, 31].

## 3. Results and Discussion

Validation of each NN model was performed to evaluate their ability to discriminate brands of fuels, using test-sets comprising the chromatograms not used in the training process. The models performance was evaluated by its classification success rate that is the percentage of correct discrimination of brands within the classified samples.

**Figure 1:**
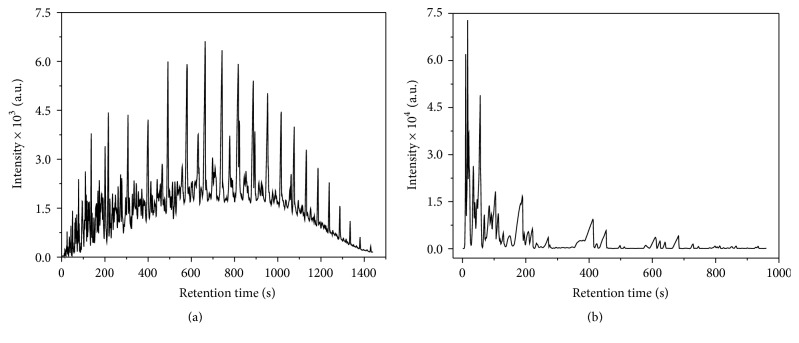
Characteristic chromatogram of (a) diesel sample and (b) gasoline sample.

Figures 1(a) and 1(b) show typical chromatogram of diesel and gasoline samples. The main difference between the chromatographic profiles is the variability in the number of the peaks. Gasoline chromatogram presents lower number of peaks due to the large amount of volatile compounds, while the diesel has a higher number of peaks corresponding to long chain hydrocarbons.

### 3.1. Data Treatment

**Figure 2:**
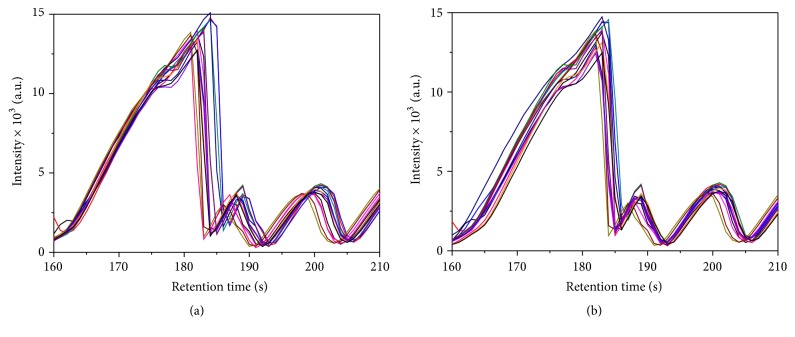
Superimposed sections of the GC-FID chromatographic trace: fifteen chromatograms of Repsol brand. (a) Before alignment. (b) After alignment.

As the first minute and the last 3 min (in diesel) and 11 min (in gasoline) of all chromatograms did not contain relevant information, these parts were removed prior to statistical analysis. In all cases, data pretreatment was necessary to correct the variations in retention time and normalization methods (relative to highest peak) were applied to take into account the injection volume and to avoid data variations. The factors that could affect the classification process are instrument variations, shifts in temperature, degradation of stationary phase, variation of pressure, and so forth [1]. Small unavoidable differences related to the instrumentation and chemical interactions between the samples and the equipment make necessary a previous alignment of chromatogram for a correct chemometric analysis [1, 3, 22, 32]. Many authors have studied the chromatogram alignment using different algorithms, but they have some difficulties such as cost-effectiveness of computer time, detection of significant peaks, or knowledge of the components in the sample [22, 33–38]. In this work, a simple and fast algorithm for alignment based on piecewise linear correlation optimized warping (COW) was used. This algorithm uses two input parameters (stretching and shrinking) estimated from the width of the peaks observed in the chromatograms [32]. The chromatograms before and after alignment for samples of Repsol brand are shown in Figures 2(a) and 2(b).

### 3.2. Classification of Diesel and Gasoline Samples

Five chromatograms were recorded for the sample from each service station. After the alignment and normalization, four chromatograms were used for training the NN model and the fifth was included in the test-set. The NN training was done using a library with 60 chromatograms (12 per brand), while the test of the NN model was done using 15 chromatograms (3 per brand) in test-set, not included in the training. The larger the information (representative data) used in the training of the NN model, the better the predictive capability of the model. Therefore, the whole set of variables that constitute the chromatogram is important in the classification process performed by the NN model.

The chromatograms used for the test-set were individually measured with respect to those used for training and unknown to the model. The NN model has the ability to classify correctly the test library of the samples used in the training. This characteristic was used as internal validation and was not usually a result with other chemometric methods (see [25]).

Despite the fact that there are not significant variations in the chromatogram to easily discriminate fuels, from the mathematical point of view, each sample can be discriminated based on its complete fingerprint. As discussed above, the possible chemical differences in the samples that allowed the identification and discrimination of the brands of fuel, could be the differences in the contents of oxygen and hydrocarbons profiles such as aromatics and olefins, producing various kinds of petroleum derivatives. Also, several studies have researched about the influence of the adding of additives, such as detergents, dispersants, antiknock agents and antioxidants, which are compounds added to improve fuel performance, avoid motor problems and minimize the amount of pollutants emitted to the atmosphere [39]. Another chemical difference that could allow such discrimination is the introduction of different trace species during the refining and blending processes that may introduce detectable differences between batches of finished fuels [8]. This fact constitutes the basis of NN ability to carry out the discrimination between the brands of fuels with high tolerance for instrument variations and the presence of outliers. Our aim is to identify exactly the point of purchase of the gasoline used in a punctual criminal act and collect samples around an established area.

In both type of samples, diesel and gasoline, all the chromatograms of each brand, included in the test of the NN model were correctly classified (3/3) over all samples, showing that the GC-NN methodology developed was able to correctly classify all of the samples to their corresponding brands.

A perfect classification was achieved for all the test chromatograms giving a 100% of success rate. In addition, no false positives or false negatives were observed in any of the models studied that shows the high robustness of methodology used. Even though the samples have been collected from different service stations, which could entail differences in transport fuel, as well as storage tanks where an external contamination is possible, NN is able to perform the classification of each of the samples in their different brands.

### 3.3. Classification of Blind Samples

In order to check the accuracy and robustness of the method, three blind samples were collected randomly from these service stations, labeled as “A,” “B,” and “C” by a third person. Three chromatograms were recorded for each blind sample and aligned with all five brands; therefore, 15 chromatograms for each blind sample were generated and used in the testing and classification process to determine the corresponding brand. Data treatment of chromatograms was carried out of the same way as reference samples.

Table 1 gives the results obtained for the chromatograms from blind samples. Although the case of sample “A” classified as BP has a low classification success rate, most of the chromatograms for this sample were assigned correctly, and only four deviated from the expected value. This behavior strongly affects the success rate, which was 73.3%. The discrepancy observed in only 4 out of 15 chromatograms not only is more than acceptable, but also is essential for taking into account the classification success rate. The “B” and “C” samples were classified correctly with 80.0% and 100% of success rate, respectively. Another important result is that no false positives or false negatives were observed. In addition, the computation time for testing each sample was below 5 s, although the test-set matrix was considerably large.

Other studies have based the classification on refineries, fuel characteristic properties, or type (normal, regular, or premium) without discrimination of brands [8, 9, 15, 16, 20]. Therefore, classifying each fuel with a specific brand, as well as performing test-set and blind sample to validate the methods, is an important factor in the results of this study.

## 4. Conclusions

A method based on gas chromatography (GC) and neural networks (NN) algorithms was developed and applied to achieve rapid identification and classification of different brands of fuels based on their characteristic fingerprints. Five brands of fuels in Spain, that is Repsol, Shell, British Petroleum (BP), Cepsa, and Galp were used. All samples analyzed were correctly classified with a high success rate. An important result was that no false positives or false negatives were observed neither in the test nor in blind samples. The combination of GC with a supervised NN method was able to perform the classification of brands due to the reliability and robustness of the estimated nonlinear classification model.

A great advantage offered by this methodology was that it did not require a detailed analysis of the fuel composition and the chromatographic fingerprint provided sufficient information to perform the classification of each brand of fuels. Also, it may provide an accurate, faster, and useful classification analysis in cases of commercial, industrial, and forensic investigations.

## Figures and Tables

**Table 1:** Neural networks classification results for blind samples.

	Blind samples	Brands of fuels					Success rate%
		Repsol	Shell	BP	Cepsa	Galp	
Chromatograms correctly classified	A	0/15	1/15	11/15	1/15	0/15	73.3
	B	0/15	0/15	0/15	0/15	12/15	80.0
	C	0/15	2/15	0/15	15/15	0/15	100
